# Effects of Rejection by a Friend for Someone Else on Emotions and Behavior

**DOI:** 10.3389/fpsyg.2019.00764

**Published:** 2019-04-09

**Authors:** Joanna Rajchert, Tomasz Żółtak, Michał Szulawski, Dorota Jasielska

**Affiliations:** ^1^Department of Applied Social Sciences, Institute of Psychology, The Maria Grzegorzewska University, Warsaw, Poland; ^2^Educational Research Institute, Warsaw, Poland

**Keywords:** comparative rejection, relationship closeness, aggression, helping, rebuilding belonging, emotions

## Abstract

Studies show that rejection increases negative affect and aggression and decreases helping behavior toward the excluder. Less is known about emotions and behavior after rejection by a friend for someone else. In two experimental studies (*N* = 101 and *N* = 169), we tested the predictions that rejection would feel worse in a close relationship but would result in less aggression and more reconnecting behavior, especially when the reasons for rejection were unknown. The results of Study 1 showed that, as expected, among acquaintances, more aggression was noted only after comparative rejection, but among strangers, aggression was also observed after rejection with no stated reason. Negative feelings toward a new acquaintance were only marginally stronger than those toward a stranger in Study 1, but Study 2 confirmed that rejection by a best friend, and especially comparative rejection by a friend, felt worse than other conditions. Study 2 also showed that reconnecting behavior was more likely to dominate over aggressive behavior between people in close relationships than between strangers. The results are discussed mostly in light of the multimotive model of rejection.

## Introduction

Research shows that people with fewer or less satisfying bonds experience more stress and hence are more likely to succumb to diseases and die younger than those with more and more satisfying bonds (Pickett and Wilkinson, 2010). Being in a relationship, however, creates a risk of being rejected, and rejection may also lead to negative consequences such as social pain (Eisenberger and Lieberman, 2004), depressed mood, hurt (MacDonald and Leary, 2005), and anger (Chow et al., 2008). Exclusion may also threaten needs of belonging, control, meaningful existence, and self-esteem (Williams et al., 2000). Exclusion also increases aggression (Warburton et al., 2006; Chow et al., 2008; Wesselmann et al., 2010; Rajchert et al., 2017) and decreases helping behavior toward the excluder but encourages more prosocial behavior toward new potential relationship partners (Maner et al., 2007). Studies have also shown that the context of exclusion, for example, the level and sequence of rejection (Buckley et al., 2004), expectations of meeting the rejecter (Maner et al., 2007), and being rejected by an in-group or out-group or by a same-sex or opposite-sex other (Bernstein et al., 2010; Wittenbaum et al., 2010; Rajchert et al., 2018), affects emotions and behavior. The presented studies also address the question of how contextual factors influence responses to rejection. We ask whether being rejected for someone else or without being given any reason as well as acquaintance or closeness with the excluder change the behavior of the excluded person toward the excluder.

The multimotive model of exclusion (Smart Richman and Leary, 2009) suggests that whether a certain motive and the resulting behavior predominate (rebuilding belonging through prosocial acts or regaining control using aggression) depends on people’s construal of the rejection event; thus, interpretation of the situation is very important. According to this model, people consider the fairness of the rejection, expectations about the relationship, the possibility for repair, the pervasiveness or chronicity of the rejection, the value of the damaged relationship, the perceived costs of the rejection, and the possibility of relational alternatives. Smart Richman and Leary (2009, p. 7) state that “when expectations of repairing the relationship are high, the relationship is highly valued, and the costs of losing the relationship are high, then people will likely be motivated to behave in prosocial ways that promote acceptance with the rejecter and use tactics that restore a sense of belonging.” It can then be assumed based on the multimotive model that relationship factors integrated in the exclusion incident, such as the closeness of relationship with the excluder or being rejected for someone else, possibly give rise to different intensities of emotions and behaviors because they direct the interpretation of the exclusion situation.

### Relationship Closeness

The multimotive model of exclusion (Smart Richman and Leary, 2009) assumes that rejection by an acquaintance might be more threatening to belonging and control needs and bring more negative feelings than rejection by a stranger. Snapp and Leary (2001) offer five reasons why rejection by a close other would be more hurtful than rejection by a stranger: (1) the consequences of relational devaluation by a close other might be very serious; (2) rejection by a close other violates positive expectation of being accepted in positive relationships; (3) people in close relationship invest substantially in it, so losing the relationship may cost more; (4) being rejected by a stranger allows for self-protecting attributions – “the rejecter does not know me”; and (5) people value the opinions of close others more than the opinions of strangers, so negative evaluation by a close other would hurt more. Although rejection by a close other is more hurtful, the multimotive model also argues that a close relationship is more valuable, and losing it would also hurt, so people would protect close and generally satisfying relationships, even in the face of rejection. This relationship-protecting motivation would show itself in less negative behavior toward a close other after rejection than toward a stranger.

Studies show that closeness (defined by Aron et al., 2004 as the inclusion of others in the representation of the self) influences levels of hormones (progesterone and oxytocin) and increases participants’ willingness to sacrifice themselves for their partner (Brown et al., 2009). However, little is known about how a relationship with a rejecter influences the emotions and behavior toward the rejecter. To the best of our knowledge, there is just one recent study on the effects of closeness on behavior after rejection. Laws et al. (2017) showed that on days when participants experienced rejection by close others, alcohol consumption increased, but on days when participants reported being rejected by acquaintances, alcohol consumption did not increase. This finding might suggest that rejection by a close other may indeed increase hurt feelings or other negative emotions and that alcohol consumption is a way of coping with those difficult emotions. However, research on emotions after rejection by a close other vs. a stranger is not consistent.

In meta-analyses of exclusion effects conducted by Blackhart et al. (2009), the emotions of participants were also not moderated by closeness to the rejecter (e.g., friend or stranger). On the other hand, ostracism in Cyberball by a romantic partner evoked more needs deprivation and less relationship satisfaction than ostracism by a stranger (Arriaga et al., 2014). Hurt feelings are also more intense when a person is rejected by a close other, such as a friend or a partner (Leary et al., 1998), than by a stranger. Leary et al. (1998) proposed that the “defense of unfamiliarity” may be used in situations where one is rejected by a stranger, so rejection by a stranger hurts less than rejection in close relationships. A stranger does not know the target of rejection, and thus, the rejection is based on external factors, not traits specific to the target. However, this study by Leary et al. (1998) was a recall study in which participants described their feelings of being hurt and the conclusions were drawn based on the content of the stories. Almost all participants recalled situations involving close others, friends, romantic partners, and family members. In another study, Snapp and Leary (2001) tested the opposite hypothesis that a more established relationship may buffer hurt feelings. They based their predictions on the assumption that when people feel valued by the other, a minor devaluation would be ignored for the sake of a good relationship. The results confirmed the hypothesis. The participants in the low familiarity condition felt more hurt than the participants in the high familiarity condition. The conclusion could be that the effects of closeness on hurt feelings may be very context specific and depend on the level of closeness but also the context influencing the perception of the exclusion as minor or serious. Thus, comparative rejection, being rejected for someone else, could also shape the effects of closeness with the rejecter on emotions and behavior.

### Comparative Rejection

A recent study by Deri and Zitek (2017) showed that being rejected for someone else, which they termed “comparative rejection” following Leary (2005), is more painful and threatens belonging more than non-comparative rejection (not being rejected for another person, instead receiving the explicit information that the rejecter preferred to be alone). Leary (2005) assumed that non-comparative rejection would be more disturbing than comparative rejection because the rejecter prefers solitude than spending time with the rejected person. Deri and Zitek (2017) suggest the opposite, that comparative rejection feels worse and that those worsened feelings are fueled by a more intense decreased sense of belonging than in non-comparative rejection. This emotional reaction occurs because people feel rejected not only by the rejecter but also by the other for whom they were rejected. They are rejected by a pack, which may increase the feelings of rejection. According to Deri and Zitek (2017), the most important factor in determining the emotional reaction to rejection is the process of explaining what happened – making sense of the exclusion incident. Similar to our own approach, they based their predictions on the Smart Richman and Leary (2009) model, which posits that people’s understandings of and reactions to rejection are closely governed by their perceptions of the implications of rejection for one’s standing in a relationship and the associated sense of belonging and exclusion.

Moreover Deri and Zitek (2017), in their fourth study, also found evidence that, by default, people react to a rejection as though it were comparative—that is, in the absence of any information about whether they have been rejected for someone or no one else, they react as negatively as if they were rejected for someone else. When participants were informed later that they were not rejected for someone else, the information attenuated participants’ initial negative reaction; however, the information that they were, in fact, rejected for someone else did not change their initial negative feelings. The above described study used a procedure in which participants were asked to imagine a break-up in a romantic relationship, so it referred to a rejection in close relationship, but their other studies referred to strangers to track the difference between comparative and non-comparative rejection. Based on the results of Deri and Zitek (2017), it might be suspected that more intense feelings of rejection lead not only to worse feelings (feeling bad, sad, angry, and upset) but also to behavioral reactions that allow dealing with those emotions, such as aggression or inhibition of positive behavior toward a rejecter.

## Study 1

The study examined whether the type of feedback (rejection with no reason, rejection for someone else, or acceptance) differently affects overall feelings, aggression and prosocial behavior toward the rejecter depending on whether the rejecter is a stranger or an acquaintance. The predictions regarding participants’ emotions and behavior were based mostly on the multimotive model of exclusion and ostracism (Smart Richman and Leary, 2009), which suggests that exclusion by a close other results in more negative emotions than rejection by a stranger because of the higher cost of losing the close relationship, among other reasons. However, a higher value of the relationship should mitigate negative behavioral reactions to exclusion to increase the chances of rebuilding the relationship in the future. Therefore, it was suspected that exclusion feedback would differently affect behavior toward the stranger and the acquaintance. When people were given a chance to get close to an excluder, the negative effects of exclusion manipulation on affect and needs would be stronger, but the negative effects for behavior would be weaker than with strangers. The exception would be a situation when the excluder preferred other relationship options. We assumed that a close relationship rejection without any stated reason for the rejection would leave space for attributions that may serve to protect the relationship. Participants may think of different, perhaps temporary, conditions that affected the rejecters’ decision. Inhibiting negative behavior would then be a relationship-protecting behavior. However, comparative rejection would signal a lower likelihood of rebuilding or saving the current relationship because the reason is revealed – the rejecter prefers someone else. If that were true, then there would be less motivation to protect the relationship and less need to inhibit negative behavior.

Hypothesis 1 stated that with acquaintances, the negative effects of comparative rejection and rejection with no reason on negative emotions would be stronger than with strangers. However, in hypothesis 2, we predicted that aggressive behavior would be lower toward an acquaintance than toward a stranger only in the rejection with no reason condition.

### Methods

#### Participants

One hundred and one university students (16 males) in psychology and sociology, aged 18–36 years (*M* = 19.85, *SD* = 2.41), participated in the study. The protocol of the study was approved by the University’s Ethical Committee. All subjects gave written informed consent in accordance with the Declaration of Helsinki before participation.

#### Materials and Procedure

First, students were told that they could take part in a study about relationship formation, which would involve working with an unknown student of the same sex randomly chosen by the experimenter. Participants who agreed to take part were asked to name all the students with whom they were familiar to avoid pairing participants with someone they already knew. The second part of the experiment took place a few days later, also during class. The experimenter announced the pairs and checked again that participants had not been paired with someone they knew. Next, participants completed the graphic Inclusion of Other in the Self Scale (IOSS) (Aron et al., 1992). They also answered two additional questions, derived from the Subjective Closeness Index (SCI) by Berscheid et al. (1989): “How close is your relationship with your partner compared to your other relationships?” and “How close is your relationship with your partner compared to other people’s relationships with their partners?” using a similar Likert scale (1 = not at all close to 7 = exceptionally close). The IOSS and SCI were chosen, as they were previously used in a validation study of the closeness generation procedure (Aron et al., 1997) that was used next. The scales were highly correlated in the Aron et al. (1997) study and were combined into a single composite to simplify the results presentation and to maximize reliability. Because the SCI measures subjective closeness, we also added one more general item, “My relationship with my partner is close,” with a seven-point Likert scale ranging from 1 (strongly disagree) to 7 (strongly agree).

##### Closeness generation procedure

The class was randomly divided into two groups. One group of students was taken to a separate room, allegedly to have more space to work in pairs. The group that left the classroom was the control group (*N* = 46). Upon arrival to the separate room, the participants in that group were instructed to sit separately from their partners and were asked to perform nine tasks and then rate how willingly they thought other participants would do each of those tasks in a different study. The tasks involved mentally completing arithmetic operations such as dividing or multiplying, reading a paragraph about animals in the jungle and answering questions, memorizing a list of five words, reading text and filling in gaps from a list of words, selecting the most interesting architectural pictures from 10 pictures presented, categorizing words (animals, tools), reading short poems and deciding which was the best, describing what they did the previous day, and remembering as many fruits and vegetables as they could that start with “p,” “a” or “c.” This activity was used as a buffer to separate the two closeness measurements and was not to be analyzed further. We used different easy tasks involving reading, counting and observing to avoid weariness but also emotional changes. The participants had 45 min to perform the tasks, and most of them finished in the time provided. While the control group participants were completing the tasks, the participants in the experimental group were instructed to sit next to their partners so that they would feel comfortable speaking privately. The paired participants answered 36 questions taken from the Aron et al. (1997) small-talk set. Each question was written on a separate small strip of paper, and the papers were clipped together in three sets of 12 strips. The participants started with the first set and answered the questions in the order in which they were given, taking turns answering first. After this, the pairs were instructed to sit as far as possible from their experimental partners. After 45 min of either the interactions or filler tasks, both groups completed four items measuring the closeness of the relationship with the partner for a second time and the acceptance-rejection procedure commenced.

##### The procedure of exclusion

The students declared whether they wanted to work on the next task with their experimental partner by marking one of three statements: (1) “I want to be in a pair with my allocated partner,” (2) “I prefer to be paired with someone else from the class,” or (3) “I definitely do not want to be paired with a partner.” They were told that their feedback would be delivered to their partner shortly afterward by the experimenter. The responses were collected from each participant and placed into one large box. However, none of the students could see which response their partner had chosen before it was handed out by the experimenter. In fact, the declared preferences (without exception, participants opted for working with their experimental partner) were swapped with randomly assigned feedback, indicating either the acceptance of the experimental partner (preference for the partner), rejection with no reason (unwillingness to be in a pair with the allocated partner) or preference for being paired with another person (comparative rejection). Afterward, participants used nine-point scales to indicate the extent to which they felt rejected (“I feel not accepted by my partner”) and felt control over the relationship (“My opinion counts to my partner”; “I have influence over my partner’s decisions”; “I have control over the relationship with my partner”), as well as their feelings of sadness, anger, hurt, and overall good mood and happiness (these last two items were aggregated into a positive affect index).

##### The aggression measurement

Next, aggression was measured using a new procedure developed by Saleem et al. (2015), called the Tangram Help-Hurt Task. The participants were informed that they would be building tangrams that would be assigned to them by their partner after they first chose puzzles for their partner. If participants managed to complete 10 out of 11 tangrams selected by their partner within 10 min, they would receive a reward. The participants chose tangrams to assign to their experimental partner from a set of 30, of which 10 tangrams were labeled ‘difficult,’ 10 were labeled ‘medium,’ and 10 ‘easy.’ The aggression score was the number of difficult tangrams assigned to the partner, while the helping score was the number of easy tangrams assigned to the partner. However, because the partner could skip one tangram, we subtracted one from the total number of allocated easy and difficult tangrams, and all negative values were transformed to zeros. In the validation studies, help and aggression scores were correlated, *r* = -0.73 (Saleem et al., 2015).

After the aggression and helping measurement, participants indicated their motivation to help or hurt their partner by responding to two questions (“I wanted to help my partner to earn the reward” and “I wanted to make it difficult for my partner to earn the reward”) using a five-point Likert scale (1 = strongly disagree to 5 = strongly agree). After completing the assessment, the participants were asked what they thought about the study and whether they had any questions, and they were debriefed and thanked.

#### Statistical Analysis

The study was a hybrid of dyadic and individual design. The closeness treatment variable seemingly was a typical dyad-level variable – both participants in one pair either had (1-1) or did not have (0–0) a structured conversation. Along with the Kenny et al. (2006) prescriptions for dyadic analysis, in such cases, partial interclass correlation (ICC) should be tested for within each pair, and if cores in pairs are correlated, the pair should be used as the subject of analysis instead of the individual. However, dyadic analysis is not well suited to the design of the present study because in the no-interaction group, partners did not have a chance to influence each other. Additionally, the rejecting or accepting feedback was presented randomly to individuals and could be the same or different within a pair. In this situation, proper statistical inference can be assured by use of robust (cluster-corrected) standard errors estimated using the sandwich method (Binder, 1983) only for the group in which scores in pairs would be correlated.

### Results

#### Preliminary Analysis

On the basis of confirmatory factor analysis, aggregated indices of closeness were created from the IOSS, SCI and one additional item measuring general closeness (Closeness 1, measured before manipulation, Cl1; Closeness 2, measured after manipulation, Cl2). The results of separate factor analyses for time 1 and time 2 showed that all four items loaded on one factor (*r*-values fell between 0.35 and 0.65 for Cl1 and 0.69 and 0.81 for Cl2).

Next, the ICC was conducted separately for small-talk and control conditions for all studied variables to test for the non-independence of the scores in the pairs. In the control group, only the baseline feeling of closeness (CL1) was correlated in the pairs (*r_I_* = 0.398, *p* = 0.026) because all the participants were strangers to each other. Thus, individual participants rather than pairs in the control group should be the objects of analysis, consistent with the suggestion of Kenny et al. (2006). The results for the small-talk group showed that the threshold of *p* < 0.20 (recommended by Kenny et al., 2006) for ICC was exceeded in the case of feelings of closeness before and after the manipulation (CL1 and CL2), aggression, helping, positive affect, sadness and hurt, despite the fact that feedback was allocated randomly to partners in the pairs. We also tested for gender differences in closeness, but participants’ sex was not related to CL1 and CL2, so sex was not included in further analysis.

In a second step of the preliminary analysis, the effectiveness of the relationship closeness manipulation was tested using repeated measures analysis of variance (RM ANOVA) with robust, cluster-corrected standard errors estimated using the sandwich method in a 2 (time of measurement: before and after closeness manipulation) × 2 (small-talk; no interaction) design. The results showed the main effect of measurement time, *F*(1,96) = 131.14, *p* < 0.001 $\eta_{p}^{2}$ = 0.25 and the interaction of measurement time and manipulation, *F*(1,96) = 135.67, *p* < 0.001, $\eta_{p}^{2}$ = 0.25 for the feeling of closeness with the partner. The *post hoc* tests with Bonferroni correction showed that the participants in the small-talk and no interaction conditions did not differ in Cl1, but the participants in the small-talk group had higher Cl2 than the participants in the control group, *p* = 0.001. Additionally, while there was no difference between Cl1 and Cl2 in the no interaction group, in the small-talk group, Cl2 was higher than Cl1. The means are presented in Table 1. These analyses confirmed that the small-talk procedure was effective in closeness generation.

**Table 1:** Means, standard deviations and 95% confidence intervals for means of Cl1 and Cl2 in small-talk and no interaction conditions.

	No interaction			Small-talk		
	*M*	*SD*	95% CI	*M*	*SD*	95% CI
Cl1 (*N* = 46; α = 0.76)	1.68	0.75	1.47; 1.90	1.35	0.54	1.21; 1.50
Cl2 (*N* = 55; α = 0.92)	1.67	0.76	1.45; 1.89	3.27	1.13	2.97; 3.57

#### Emotions

Scores for negative emotions (anger, hurt, and sadness) and positive emotions (happiness and feeling good) were correlated, so they were aggregated into negative and positive emotions scales. Hypothesis 1 posited that in the small-talk condition, the negative effects of comparative rejection and rejection with no reason on feelings would be more negative than with strangers. To test this prediction, a two-factor ANOVA was conducted with feelings of rejection, control, and emotions as dependent variables and feedback (acceptance; rejection with no reason; comparative rejection) and closeness (small-talk, no interaction) as independent variables. Because sex was not related to positive and negative emotions, it was not included in the model.

The results showed that feedback influenced feelings of rejection, *F*(2,96) = 13.49, *p* < 0.001, $\eta_{p}^{2}$ = 0.21; control, *F*(2,96) = 6.44, *p* = 0.003, $\eta_{p}^{2}$ = 0.12; positive emotions, *F*(2,96) = 8.25, *p* < 0.001, $\eta_{p}^{2}$ = 0.12; and negative emotions, *F*(2,96) = 11.05, *p* < 0.001, $\eta_{p}^{2}$ = 0.19. The mean feelings of rejection, control and emotions are presented in Table 2.

**Table 2:** Means, standard deviations (M/SD) and 95% confidence intervals [95% CI] of feelings after acceptance, rejection with no reason, and comparative rejection.

			Comparative
Variable	Acceptance	Rejection	rejection
Variable	*M/SD* [95% CI]	*M/SD* [95% CI]	*M/SD* [95% CI]
Rejected	2.00/2.46 [1.08; 2.91]	5.39/3.01 [4.36; 6.40]	4.22/2.37 [3.41; 5.04]
Control	4.00/1.96 [3.27; 4.74]	3.08/1.53 [2.56; 3.60]	2.72/1.25 [2.29; 3.15]
Positive emotions	7.30/1.52 [6.72; 7.87]	5.56/2.42 [4.75; 6.38]	5.68/2.27 [4.90; 6.46]
Negative emotions	1.34/0.72 [0.78; 1.90]	3.14/1.98 [2.57; 3.60]	2.67/1.70 [2.11; 3.15]

The *post hoc* test with Bonferroni correction showed that participants felt more rejected and more negative emotions and less control and positive emotions after rejection with no reason and after comparative rejection than after acceptance (*p*-values were between <0.001 and 0.037). There were no significant differences in feelings between rejection with no reason and comparative rejection conditions.

The main effect of closeness was observed for control, *F*(2,96) = 11.53, *p* < 0.001, $\eta_{p}^{2}$ = 0.10. The participants in the small-talk conditions declared greater feelings of control, *M* = 3.62, *SD* = 1.67, 95% CI [3.17; 4.08] than those in the comparison group, *M* = 2.76, *SD* = 1.57, 95% CI [2.30; 3.21]. Additionally, negative emotions were lower among participants who were strangers with their partners, *M* = 2.09, *SD* = 1.31, 95% CI [1.64; 2.54], than among those who were acquaintances, *M* = 2.62, *SD* = 1.94, 95% CI [2.20; 3.04], but the effect was only marginally significant, *F*(2,96) = 2.95, *p* = 0.089, $\eta_{p}^{2}$ = 0.03 (below *p* = 0.10). The only significant interaction also referred to control, *F*(2,96) = 4.36, *p* = 0.017, $\eta_{p}^{2}$ = 0.12. Feelings of control were higher after receiving acceptance feedback than after receiving rejection and comparative rejection feedback (both *p*-values < 0.001), but only in the small-talk group (the means are presented in Table 3). The results also indicated that participants felt more control when they were accepted after the small-talk than when they were accepted without the opportunity to talk with their partners (*p* = 0.002).

**Table 3:** Means, standard deviations and 95% confidence intervals of control in small-talk and no interaction condition after different feedback.

Feedback	No interaction			Small-talk		
	*M*	*SD*	95% CI	*M*	*SD*	95% CI
Acceptance	2.86	1.82	2.11; 3.62	5.15	1.34	4.39; 5.90
Rejection with no reason	2.88	1.58	2.12; 3.64	3.22	1.52	2.58; 3.86
Comparative rejection	2.54	1.21	1.81; 3.28	2.86	1.30	1.81; 3.28

#### Behavior

First, we examined whether aggression and prosocial behavior were motivated by a desire to make it more difficult or easier for one’s experimental partner to earn the reward. Correlation analysis showed that the aggression score was positively related to the intention to hinder one’s partner’s chances for the reward (*r* = 0.69, *p* < 0.001) and negatively related to the intention to help one’s partner receive the reward (*r* = -0.60, *p* < 0.001). Correspondingly, the helping score was positively related to the intention to help one’s partner (*r* = 0.67, *p* < 0.001) and negatively related to the intention to hinder the chance for the reward (*r* = -0.61, *p* < 0.001). Helping and aggression scores were also negatively correlated (*r* = -0.71, *p* < 0.001). Sex was not related to behavior, so it was not included in further analysis.

**FIGURE 1:**
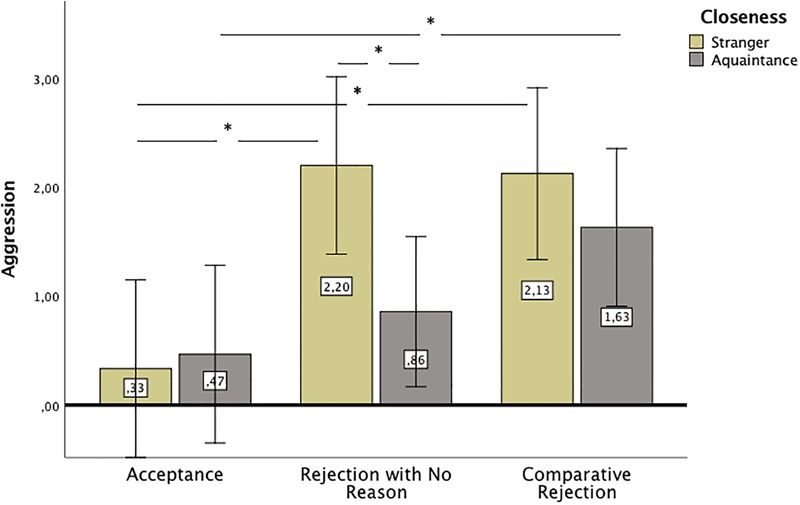
Mean aggression in no interaction (stranger) and small-talk (acquaintance) conditions after different feedback. Error bars represent 95% CI. Significant differences between groups are marked with asterisk.

The second hypothesis predicted that aggressive behavior would be lower toward an acquaintance than toward a stranger only in the rejection with no reason condition. We used two-way ANOVA (with robust, cluster-corrected standard errors estimated using the sandwich method) to explore the interactive effect of closeness induction and rejection on aggression and helping. There was a main effect of the feedback on aggression, *F*(2,96) = 12.44, *p* < 0.001, $\eta_{p}^{2}$ = 0.14. The participants in the rejection condition, *M* = 1.53, *SD* = 1.84, 95% CI [0.99; 2.06], and the comparative rejection condition, *M* = 1.88, *SD* = 1.94, 95% CI [1.34; 2.41], indicated higher aggression than the accepted participants, *M* = 0.40, *SD* = 0.62, 95% CI [-0.18; 0.98], Bonferroni corrected *p* = 0.004 and *p* = 0.001, respectively. The participant groups who were rejected with no reason and rejected for someone else did not differ in aggression. The main effect of closeness (small-talk, control) approached significance, *F*(2,96) = 3.00, *p* = 0.087, $\eta_{p}^{2}$ = 0.03. Aggression was higher in the no interaction group, *M* = 1.55, *SD* = 2.16, 95% CI [1.09; 2.02], than in the small-talk group, *M* = 0.98, *SD* = 1.17, 95% CI [0.55; 1.41]. Additionally, the interaction between closeness and feedback approached the significance threshold, *F*(2,96) = 2.655, *p* = 0.077, $\eta_{p}^{2}$ = 0.03. The hypothesis concentrated mainly on the differences between aggression toward acquaintances and strangers in the case of rejection with no reason. *Post hoc* tests confirmed the hypothesis (although without Bonferroni correction). In the rejection with no reason condition, aggression toward a stranger was higher than toward an acquaintance, *p* = 0.038, but in the comparative rejection condition, there was no difference between the no interaction and small-talk conditions, *p* > 0.48. Other *post hoc* tests indicated that in the small-talk condition, only comparative rejection and acceptance feedback differed in aggression, *p* = 0.002. The participants who were rejected for someone else by an acquaintance chose more difficult tangrams for the rejecter than the participants who were accepted by an acquaintance. However, in the no interaction condition, differences in aggression emerged not only between acceptance and comparative rejection feedback, *p* = 0.007, but also between acceptance and rejection with no reason feedback, *p* = 0.004. There were no significant differences between comparative rejection and rejection with no reason in the no interaction group. The mean aggression scores in the small-talk and no interaction groups and conditions are presented in Figure 1.

Analysis of helping with the same independent variables showed a main effect only of feedback, *F*(2,96) = 5.56, *p* = 0.005, $\eta_{p}^{2}$ = 0.09, with the participants in the comparative rejection condition helping less, *M* = 3.29, *SD* = 2.63, 95% CI [2.41; 4.18], than the accepted participants, *M* = 5.23, *SD* = 2.20, 95% CI [4.28; 6.19], *p* = 0.011. Differences in helping between the rejected, *M* = 3.77, *SD* = 2.87, 95% CI [2.89; 4.65] and accepted participants were also significant, *p* = 0.046, with the participants who were rejected with no reason helping less than those who were accepted. Helping in the rejection and comparative rejection conditions was not different.

Although no hypothesis was formulated for this topic, whether feelings of closeness measured after the small-talk or filler tasks in the control group moderated the rejection effect on aggression and helping was also tested. The results of a series of regression analyses showed no significant interaction between Cl2 and feedback.

### Discussion

The present study was designed to verify two predictions formulated based on the Smart Richman and Leary (2009) multimotive model. A novel and central element in this study was two situational factors that shaped exclusion effects on emotions and behavior, namely, relationship closeness with a rejecter and comparative rejection (being rejected for someone else). We predicted that a positive relationship with the rejecter will make rejection feel worse but, at the same time, will restrain aggressive tendencies, but only when the reasons for rejection are not known.

In general, the results showed more distress (more negative and less positive feelings) in both the rejection and comparative rejection conditions compared to acceptance. This result is in line with the results of many other studies (Gerber and Wheeler, 2009). Moreover, the results also showed that comparative rejection did not feel worse than rejection with no reason, which conforms with Deri and Zitek (2017) study showing that only rejection for no one else (non-comparative rejection) resulted in less negative feelings. However, in hypothesis 1, we predicted that negative affect should be stronger after rejection by an acquaintance than after rejection by a stranger. The results showed that the differences were in line with this hypothesis in the case of negative affect, but the effect was only marginally significant. The result considering negative emotions was also consistent with a recall study by Leary et al. (1998) showing that hurt feelings after rejection increase with the closeness of the relationship. Closeness generation also affected feelings of control, but this could be interpreted mainly as an increase in control after being accepted by an acquaintance, rather than a decrease in control due rejection.

Considering behavior, an interesting pattern of results emerged. The participants in comparative rejection conditions were as aggressive and helpful as those participants who were rejected with no reason but more aggressive and less helpful than accepted participants. Thus, the general picture was that comparative rejection did not result in higher aggression and lower helping than rejection with no reason (which is in line with the results for emotions). However, this pattern of differences in aggression holds only among strangers. In hypothesis 2, we predicted that when the rejecter is a close other and the reason for the rejection is not clearly stated, then more relationship-protecting behavior and less aggression will occur than when the rejecter is a stranger. The results confirmed this hypothesis, although, as in the case of hypothesis 1, the effects were only marginally significant. Additionally, compared to participants in the acceptance condition, acquaintances reacted with more aggression only in the comparative rejection condition and not in the rejection with no reason condition, which is an additional support for the hypothesis. For people who became acquainted with their partner, who thought that they had something in common, and who created positive expectations regarding the relationship, the information that the partner preferred other relationships resulted in the highest aggression.

In sum, Study 1 yields results that are in line with the multimotive model of exclusion and our hypothesis, but the effects are very small and should be interpreted with caution. Thus, we decided to conduct another study to verify our hypothesis.

## Study 2

Study 2 tested the same two hypotheses as Study 1 but used different designs and procedures. Most importantly, only two rejection conditions were compared (there was no acceptance condition), and the study did not test actual behavior but rather likely behavior or probable emotions in the rejection scenario. Thus, the results of this study may differ from Study 1, in the same way that intended or planned behavior may differ from actual behavior (Madden et al., 1992). We also asked participants about tendencies for reconnection (behaviors that rebuild a lost sense of belonging). We assumed that comparative rejection yields fewer possibilities for relationship-protecting attributions of the reasons behind rejection than rejection with no reason. Comparative rejection is also more final, so it offers fewer chances for rebuilding or developing the relationship. Additionally, reconnection tendencies should be more intensive toward friends because of the more negative consequences of losing valued relationships. Thus, in hypothesis 3, we predicted that behaviors rebuilding belonging would be more likely toward friends than toward strangers, and in hypothesis 4, we predicted that reconnecting behaviors would also be less likely after comparative rejection than after rejection with no reason, but (5) the difference in reconnecting behavior between comparative rejection and rejection with no reason would be stronger among friends.

Participants in each group were asked to imagine themselves being in two situations. In the first situation, they were rejected by a close friend, whereas in the second situation, they were rejected by a stranger. Moreover, one group of participants imagined that a friend and a stranger preferred someone else over them, but the other group did not receive any reason for rejection.

### Methods

#### Participants

The participants were 169 students (16 men) aged 19–29, *M* = 21.88, *SD* = 2.29. They volunteered for the study and were not compensated for their participation.

#### Materials and Procedure

The study was conducted in groups of 4–20 people. The participants were asked to read descriptions of two situations: the first involved rejection by a friend (“*After leaving for a vacation, you send your best friend pictures from your trip and messages online. You ask what he/she is up to. The friend replies: ‘Listen, I do not want to talk to you, and I do not want to be friends with you anymore.”’*); the second involved rejection by a stranger *(“It is the beginning of new academic year. You are going to your first class, to a new group of people, where you do not know anyone. You are to work in pairs. The teacher sends you a list of topics, and you are to put your name by the topic you would like to work on. You choose one topic and see that somebody else also chose this topic. You are supposed to work in pairs, so you write an e-mail to this person asking how you should split your work. The person replies: ‘I do not want to work with you”’*). One group also read additional information about the case of rejection by a friend (*“At the same time, on social media, your friend is posting clips and pictures having great time with people who you both used to hate.”*) and the case of rejection by a stranger: *“I want to work with someone else – we already agreed.”*). The participants in this group (*N* = 87, 8 men) were in the comparative rejection condition. The participants who did not obtain information that the rejecter preferred someone else were in the rejection with no reason group (*N* = 82, 8 men). Below the description of each situation, there were questions asking the participants, “How likely is it that you would feel angry, hurt, disturbed, or rejected; ask the person about the reason for the behavior; write or say how hurt you are; send repeated messages; write or say nasty or insulting things to the person; want to push or hit the person; want to yell at the person; want to hit or destroy something; worry about whether the person is all right; or think that you still can do something in this situation.” The questions referred to negative emotions, feelings of rejection and behavioral tendencies that were aggressive and aimed at reconnecting with the rejecter (rebuilding of a sense of belonging). Participants used a five-point scale (1 – not likely at all; 5 – very likely) to answer those questions.

### Results

First, we conducted exploratory factor analysis with all of the questions, which showed three factor solutions. Anger, hurt and rejection loaded on one factor, which was named “negative feelings,” Cronbach α = 0.71, for scenarios with a friend and α = 0.81 for scenarios with a stranger. Behavioral intentions for reconnection (asking the person about the reason for the behavior, writing or saying how hurt you are, sending repeated messages, worrying about whether the person is all right, thinking that you still can do something in this situation) loaded on the second factor, α-values = 0.76, for scenarios both with a friend and with a stranger. The aggressive behavior factor included writing or saying nasty or insulting things to the person, wanting to push or hit the person, wanting to yell at the person, and wanting to hit or destroy something, α = 0.761 for scenarios with a friend and α = 0.85 for scenarios with a stranger. Aggressive tendencies were related to negative emotions among friends (*r* = 0.36, *p* < 0.001) and among strangers (*r* = 0.31, *p* < 0.001). In the case of strangers (but not friends), reconnection tendencies were also related to negative emotions (*r* = 0.37, *p* < 0.001). This pattern of correlations was the same in the comparative rejection and rejection with no reason groups. Although only 16 men participated in the study, we tested whether they differed from women in negative feelings and behavioral tendencies. Results showed that women had higher tendency than men for reconnection with friends, *t*(1,167) = 3.03, *p* = 0.003, *d* = 0.79, and with strangers, *t*(1,167) = 2.07, *p* = 0.040, *d* = 0.56 (reconnection with friends: *M*_women_ = 3.77, *SD* = 0.85, *M*_men_ = 3.08, *SD* = 0.90; reconnection with strangers: *M*_women_ = 2.04, *SD* = 0.84, *M*_men_ = 1.58, *SD* = 0.79). Women also declared more negative feelings than men after rejection by a friend, *t*(1,167) = 2.36, *p* = 0.019, *d* = 0.58, and after rejection by a stranger, *t*(1,167) = 2.40, *p* = 0.017, *d* = 0.58 (rejection by a friend: *M*_women_ = 4.17, *SD* = 0.79, *M*_men_ = 3.66, *SD* = 0.96; rejection by a stranger: *M*_women_ = 3.56, *SD* = 1.04, *M*_men_ = 2.89, *SD* = 1.23). There were no sex differences in aggressive tendencies after rejection (*p* > 0.26).

#### Negative Feelings

**FIGURE 2:**
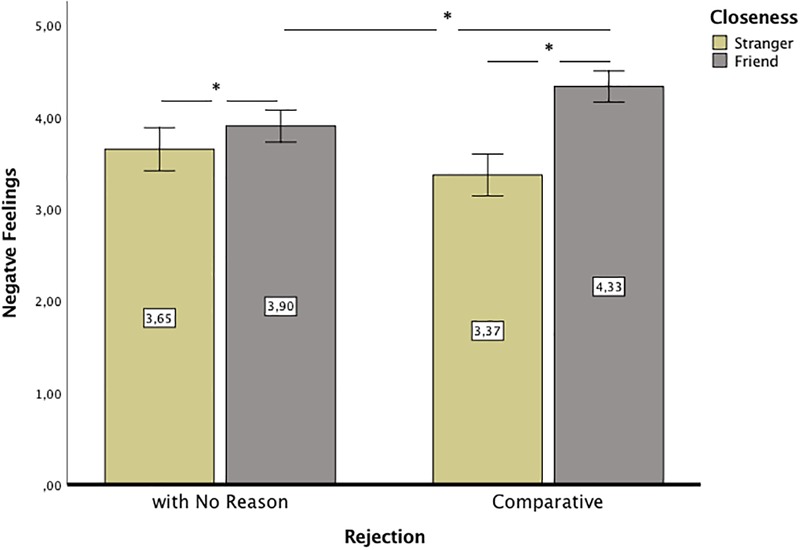
The mean negative feelings after comparative rejection and rejection with no reason by a friend and a stranger. Error bars represent 95% CI. Significant differences between groups are marked with asterisk.

Next, RM ANOVA with one within-participant variable, i.e., closeness (friend vs. stranger), and one between-participant variable, i.e., group (comparative rejection vs. rejection with no reason), were conducted for negative feelings. As men and women differed in negative feelings, sex was included in the analysis as a covariate, but it did not differentiate the effect of closeness (friend vs. stranger) on negative feelings, *p* > 0.53. Negative feelings were higher after rejection by a friend, *M* = 4.12, *SE* = 0.06, 95% CI [3.99; 4.24], than after rejection by a stranger, *M* = 3.51, *SE* = 0.08, 95% CI [3.34; 3.67], *F*(1,166) = 45.02, *p* < 0.001, $\eta_{p}^{2}$ = 0.21. This result confirmed hypothesis 1. The group did not differentiate negative affect (*p* > 0.54), but the interaction of group and closeness did, *F*(1,166) = 18.09, *p* < 0.001, $\eta_{p}^{2}$ = 0.10, which was not predicted. The effect of this interaction remained similar when sex was not controlled in the model, *F*(1,167) = 18.10, *p* < 0.001, $\eta_{p}^{2}$ = 0.10. The *post hoc* test revealed that negative feelings were higher when the rejecter was a friend than when the rejecter was a stranger, and the difference was significant in the comparative rejection condition, *p* < 0.001, $\eta_{p}^{2}$ = 0.29 as well as in the rejection with no reason condition, *p* = 0.038, $\eta_{p}^{2}$ = 0.03; however, in the comparative rejection condition the effect was much stronger than in the rejection with no reason condition. Additionally, participants indicated more negative feelings after comparative rejection by a friend than after rejection with no reason by a friend, *p* = 0.001, $\eta_{p}^{2}$ = 0.07, but the difference was not significant when rejecter was a stranger, *p* > 0.08, $\eta_{p}^{2}$ = 0.02. The mean negative feelings in conditions is presented in Figure 2.

#### Behavior

**FIGURE 3:**
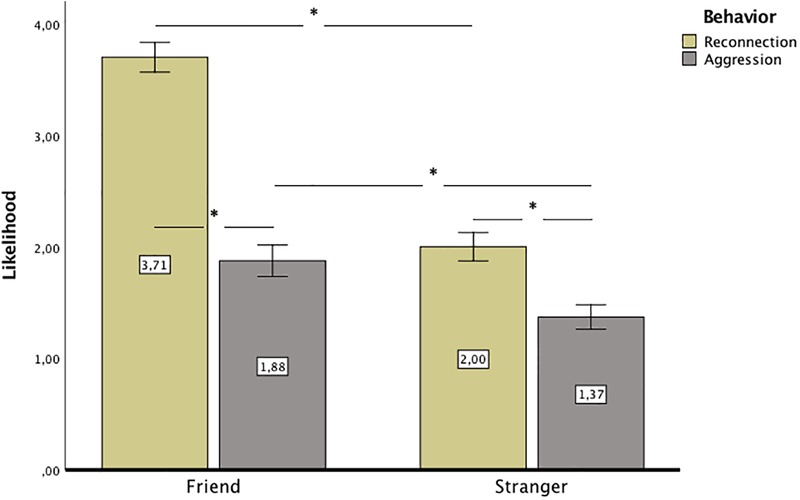
Mean reconnecting and aggressive behavior after rejection by a friend and by a stranger. Error bars represent 95% CI. Significant differences between groups are marked with asterisk.

Next, we verified predictions regarding behavior. An RM ANOVA was conducted with group (comparative rejection or rejection with no reason) as the between subject variable and two within subject variables, i.e., likelihood of a certain behavior (aggressive or reconnecting) and closeness (friend or stranger). Sex was included as a covariate in the analysis because it was related to reconnecting tendencies. The results showed that group did not differentiate behavior, *p* > 0.10, but the probability of any behavior (aggressive or reconnecting) toward a friend was larger, *M* = 2.79, *SE* = 0.05, 95% CI [2.70; 2.88], than toward stranger, *M* = 1.69, *SE* = 0.04, 95% CI [1.60; 1.77], *F*(1,165) = 360, *p* < 0.001, $\eta_{p}^{2}$ = 0.69. This effect was not different between the groups rejected for someone else or with no reason, *p* > 0.19 or between men and women, *p* > 0.88. The analysis indicated that, in general, reconnecting behavior was more probable, *M* = 2.85, *SE* = 0.05, 95% CI [2.75; 2.96], than aggressive behavior, *M* = 1.62, *SE* = 0.05, 95% CI [1.52; 1.73], *F*(1,165) = 265.63, *p* < 0.001, $\eta_{p}^{2}$ = 0.62. This effect was moderated by sex, *F*(1,165) = 8.66, *p* = 0.004, $\eta_{p}^{2}$ = 0.05. Both, men and women had greater tendency for reconnecting behavior, *M*_men_ = 2.33, *SE* = 0.16, 95% CI [2.01; 2.66], *M*_women_ = 2.90, *SE* = 0.05, 95% CI [2.80; 3.01], than for aggressive behavior, *M*_men_ = 1.79, *SE* = 0.17, 95% CI [1.44; 2.14], *M*_women_ = 1.60, *SE* = 0.06, 95% CI [1.49; 1.72], but the effect was much stronger for women, *p* < 0.001, $\eta_{p}^{2}$ = 0.61, than for men, *p* = 0.031, $\eta_{p}^{2}$ = 0.03. Difference between tendency for reconnecting and aggressive behavior only marginally varied between groups rejected for someone else or for no reason, *F*(1,165) = 3.52, *p* = 0.06, $\eta_{p}^{2}$ = 0.02; the difference between comparative rejection and rejection with no reason was significant in the case of reconnection, *p* = 0.010, $\eta_{p}^{2}$ = 0.04, but not aggression, *p* > 0.83. Reconnection was more likely in the rejection with no reason condition, *M* = 2.98, *SE* = 0.0.07, 95% CI [2.84; 3.13], than in the comparative rejection condition, *M* = 2.72, *SE* = 0.07, 95% CI [1.48; 1.79], but there was no such difference in case of aggression (*M* = 1.63 in the comparative rejection condition and *M* = 1.61 in the rejection with no reason condition). This last result confirms hypothesis 4, which argued that reconnecting behaviors would be less likely after comparative rejection than after rejection with no reason. The likelihood for a particular behavior also differed depending on closeness, *F*(1,165) = 135.12, *p* < 0.001, $\eta_{p}^{2}$ = 0.45. Although the prevalence of reconnecting behavior was observed in friends, *p* < 0.001, $\eta_{p}^{2}$ = 0.66 as well as in strangers, *p* < 0.001, $\eta_{p}^{2}$ = 0.27, the effect was twice as strong in friends than in strangers. Additionally, the difference in reconnecting behavior toward friends and strangers was significant, *p* < 0.001, $\eta_{p}^{2}$ = 0.72, which was in line with hypothesis 3 that behaviors to rebuild belonging would be more likely toward friends than toward strangers. The difference in aggression toward friends and strangers was also significant, *p* < 0.001, $\eta_{p}^{2}$ = 0.26, but the difference was larger in the case of reconnecting behavior, indicating that the motivation for re-establishing belonging is the main differentiating factor in behavior toward friend vs. stranger. The interaction of closeness and type of behavior was not different between men and women, *p* > 0.25. The means for reconnecting and aggressive behaviors after rejection by a friend and by a stranger are presented in Figure 3.

The results also showed that closeness × type of behavior × group interaction was not significant, *p* > 0.50 (the effect remained insignificant when sex was extracted from the model). Thus, hypothesis 2 stating that aggressive behavior would be lower toward a close other than toward a stranger only in the rejection with no reason condition, and hypothesis 5 stating that the difference in reconnecting behavior between comparative rejection and rejection with no reason would be stronger among friends, were not confirmed.

### Discussion

The objective of Study 2 was to verify the hypothesis already tested in Study 1 that negative feelings would be stronger among friends than among strangers (hypothesis 1) and that aggressive behavior would be stronger among strangers than among friends but only after rejection with no reason. The first hypothesis was confirmed in Study 2, but the second was not. The results showed that, as expected, when people were asked to imagine their emotional reactions to rejection, they thought that they would feel worse when rejected by a friend than when rejected by a stranger. This result is in line with the multimotive model of exclusion (Smart Richman and Leary, 2009) as well as with results of studies showing more hurt feelings when recalling rejection by a close other (Leary et al., 1998) and more threats to needs after ostracism (Arriaga et al., 2014). Regarding the interactive effect of closeness and comparative rejection on negative emotions, the results showed that feelings were more negative after comparative rejection than after rejection with no reason only when the rejecter was a friend. This result is not entirely in line with a previous study using an imagination procedure (Deri and Zitek, 2017), which showed no differences in emotions between being rejected by a romantic partner for someone else and being rejected with no reason. In Study 2, this was true only in the case of strangers. Differences in the results might be due to the different scenarios that we used and non-romantic relationships with the rejecter. Rejection with no reason in romantic relationships might feel worse than in friendly relations, making it more similar in emotional effects to comparative rejection. Additionally, the scenario that we used might be less credible and thus less hurtful without information about the friend having fun with a new attractive pack of friends.

Our second hypothesis regarded aggressive behavior. Contrary to our expectations, we found a general tendency for more aggression toward a close other than toward a stranger.

Study 2 also showed that although people felt worse and more aggressive toward friends than toward strangers, they were also more likely to provide an opportunity for rebuilding the relationship with close others than with distant others. They were more likely to believe that they could change the situation by sending messages or expressing their feelings toward friends than toward strangers. The multimotive model (Smart Richman and Leary, 2009) assumes that both motivations – to rebuild the relationship and to regain control thwarted by rejection – might be evoked simultaneously. In this model, motivation for regaining belonging is related to more prosocial actions, while motivation for rebuilding control is related to more aggressive behavior. In our study, as the multimotive model posits, participants believed that after rejection, they were capable of both aggression and positive behavior. However, in the case of behavior toward friends, rebuilding belonging dominated over aggressive tendencies, even though people were more hurt and angry than when rejected by a stranger. The likelihood for reconnecting behavior was also higher than that for aggression in the case of strangers, although the effect was much weaker than in the case of friends. The results regarding the prevalence of reconnecting behavior in friends, although not directly predicted, provide a more general look at the studied matter. They show that in close relationships, emotions and reconnecting behaviors, as well as aggressive behaviors, are more intense because the relationship is more important and losing it could have devastating consequences. However, relationship-protecting behaviors would predominate in interactions with friends even in the face of rejection, while this possibility is less evident in relationship with strangers. It is also probable that women manifest more positive behaviors, and experience stronger negative feelings toward those who rejected them (both friends and strangers) than men. Although this result should be treated with a great caution as we gathered data only from 16 men, it would be in line with recent findings (Rajchert et al., 2018) that men are more aggressive and less helpful after rejection than women.

## General Discussion

Based on the study results, we can conclude that the multimotive model as well as the temporal need-threat model (Williams, 2009), correctly posited that the immediate reaction to any sign of exclusion is the feeling of being rejected, sadness, anger, less positive affect and pain (hurt feelings). Moreover, both of the present studies indicate that rejection for someone else or rejection with no reason results in similar intensity of negative emotions, which is in line with results of the study by Deri and Zitek (2017). However, studies also show that when rejection happens in a very close relationship, it elicits more negative feelings than when it happens with a stranger, although this effect in Study 1 was very small. Perhaps an acquaintance is not a close enough relationship to make a substantial difference in negative feelings. It may also be that what people think they would feel is not what they truly feel in a particular situation.

The exclusion models also propose that behavioral reactions might shift depending on the individual interpretation of the situation, which may be affected by the exclusion context. The results presented here confirm that the social context of rejection influences behavior. However, the results of the studies do not seem consistent. Whereas Study 1 shows that getting close to rejecter results in lowered aggression when there is no reason for rejection, Study 2 indicates that aggression toward a close other is more likely than aggression toward a stranger, regardless of whether rejection is or is not for someone else. This finding might suggest that people would be more aggressive toward rejecters who are their friends than rejecters who are strangers. Nevertheless, this might be a false conclusion considering further the results of Study 2 showing that positive behavior was much more probable toward a friend than toward a stranger after rejection. From this point of view, the results in Studies 1 and 2 are not exactly opposite; although in the case of acquaintances, a decrease in aggression is observed after rejection with no reason, while in the case of friends, there is a general tendency for reconnecting compared to aggressive behavior. In both studies, aggression is less likely when rejection occurs by a closer than a less close other, and the reasons behind the rejection are less important determinants of behavior.

### Limitations and Future Research

Although, in general, the results conformed to our expectations, more research is still needed. Both studies should be replicated with more equal sex distribution, as the samples mostly included females. This might be especially important limitation as there were sex differences in emotions and behavior in Study 2 showing more negative feelings and tendency for reconnecting behavior after rejection among women. This effect might be explained, for example, in light of tend-and-befriend theory (Taylor, 2006), which posits that tending and befriending behavior as a reaction to social rejection and other social stressors is motivated by affiliative needs and oxytocin levels. Although those reactions refer to both men and women, Stroud et al. (2002) propose that women have stronger affiliative responses to stress than men, because selection pressures for social responses to threat that benefit both self and children were greater for women than for men. Another important limitation is the weakness of some of the effects that were reported. Thus, the replication of Study 1 should include not only more men but also more participants in general.

Another limitation refers to the closeness-generating procedure in Study 1. While the procedure successfully escalated the feelings of closeness, which were higher in the small-talk group than in the control group, the feelings of closeness after the procedure remained relatively low (average of 3 out of 7). As a consequence, the word “closeness” could be misleading in case of Study 1, as one might associate it with partners who have a deep and very close relationship, such as married couples or friends. In this last case, however, other factors inherited in the relationship, such as its quality, security or intrusiveness, should be considered as factors that could change reactions to rejection. Further studies might then include couples or friends. In Study 2, on the other hand, participants only predicted their behavior and emotions toward their friends, so their actual behavior and emotions remain unknown. We can only suspect that their predictions might be based on previous experience in similar situations and their self-knowledge. In this way, we can assume that the results of Study 2 are related to real behavior. It would also be interesting to test which emotions and behaviors are experienced after comparative rejection (vs. rejection with no reason) by a friend vs. a stranger in a recall study. We could have used this procedure in Study 2 but instead decided to use scenarios because, in recall study, there is little control over what experiences participants describe; the seriousness of the recalled incident and closeness with the rejecter might vary. Participants also might have trouble making themselves remember more difficult situations. Additionally, people reconstruct their autobiographical memories (Conway and Pleydell-Pearce, 2000), so their actual reactions might have differed from what they recall during the study.

## Conclusion

Despite their limitations, current studies confirm predictions stated on the basis of a multimotive model that any, even minimal, created relationships, might influence behavioral reactions after rejection, for example, by limiting aggressive behavior or increasing prosocial behavior. We also showed that although people believe that they would feel worse after being rejected by a friend, positive behaviors toward a friend in this situation are in fact more probable than aggression. It is worth noting, however, that rejection allowing for negative social comparison (rejection for someone else) might elicit more negative fillings and aggression in close relationships than rejection with no reason. Knowing that others are more preferred is a “sting” leading to aggressive tendencies even against the befriended rejecter. However, to understand these effects more fully, more research should also be conducted with different levels of relational closeness.

## Ethics Statement

This study was carried out in accordance with the recommendations of the Declaration of Helsinki with written informed consent from all subjects. All subjects gave written informed consent in accordance with the Declaration of Helsinki. The protocol was approved by the “Ethical Committee of The Maria Grzegorzewska University.”

## Author Contributions

JR contributed to the conception and design of the study, organized the database, wrote the first draft of the manuscript, and later versions, conducted part of statistical analysis. TŻ performed part of the statistical analysis. MS and DJ wrote sections of the manuscript. All authors contributed to manuscript revision, read, and approved the submitted version.

## Conflict of Interest Statement

The authors declare that the research was conducted in the absence of any commercial or financial relationships that could be construed as a potential conflict of interest.
